# Semantic-Based Building Extraction from LiDAR Point Clouds Using Contexts and Optimization in Complex Environment

**DOI:** 10.3390/s20123386

**Published:** 2020-06-15

**Authors:** Yongjun Wang, Tengping Jiang, Min Yu, Shuaibing Tao, Jian Sun, Shan Liu

**Affiliations:** 1 Key Laboratory of Virtual Geographic Environment, Ministry of Education, Nanjing Normal University, Nanjing 210093, China; 171302144@stu.njnu.edu.cn (S.T.); 09332@njnu.edu.cn (J.S.); 2 Jiangsu Center for Collaborative Innovation in Geographical Information Resource Development and Application, Nanjing 210023, China; 3 State Key Laboratory Cultivation Base of Geographical Environment Evolution, Nanjing 210093, China; 4 State Key Laboratory of Information Engineering in Surveying, Mapping and Remote Sensing, Wuhan University, Wuhan 430079, China; yumin_ym@whu.edu.cn; 5 State Key Laboratory of Marine Environmental Science, Xiamen University, Xiamen 361005, China; 22320180155033@stu.xmu.edu.cn

**Keywords:** LiDAR point cloud, building extraction, super-points, features selection, optimized neighborhood, MRF

## Abstract

The extraction of buildings has been an essential part of the field of LiDAR point clouds processing in recent years. However, it is still challenging to extract buildings from huge amount of point clouds due to the complicated and incomplete structures, occlusions and local similarities between different categories in a complex environment. Taking the urban and campus scene as examples, this paper presents a versatile and hierarchical semantic-based method for building extraction using LiDAR point clouds. The proposed method first performs a series of preprocessing operations, such as removing ground points, establishing super-points and using them as primitives for subsequent processing, and then semantically labels the raw LiDAR data. In the feature engineering process, considering the purpose of this article is to extract buildings, we tend to choose the features extracted from super-points that can describe building for the next classification. There are a portion of inaccurate labeling results due to incomplete or overly complex scenes, a Markov Random Field (MRF) optimization model is constructed for postprocessing and segmentation results refinement. Finally, the buildings are extracted from the labeled points. Experimental verification was performed on three datasets in different scenes, our results were compared with the state-of-the-art methods. These evaluation results demonstrate the feasibility and effectiveness of the proposed method for extracting buildings from LiDAR point clouds in multiple environments.

## 1. Introduction

Building objects management is of great importance for many applications in various fields, including city planning, energy analysis, 3D reconstruction and visualization, etc. As a significant requirement of smart cities, building extraction from various remote sensing data plays an increasingly critical role in the aforementioned applications. In particular, automatic or semi-automatic building extraction algorithms from images have been extensively studied in the past. However, image distortions caused by camera lens limit the accuracy and these approaches labor-intensive, time consuming, and costly in a poor condition. Light detection and ranging (LiDAR) technology has developed rapidly in recent years. It can rapidly acquire point clouds of the urban scenes along roads with high flexibility, detailed information and precision, providing a promising and feasible method for data collection. Many relevant studies have been published over the past few decades. Compared with other types of remote sensing data, extensive studies have suggested methods to extract building from LiDAR point clouds. Existing methods can be classified into object-oriented and classification-based building extraction.

Accurate definition of objects can improve efficiency and provide accurate information for object identification. Object-based methods have therefore been proposed and the general idea is to detect segments with the use of algorithms like region growing. In the early years, building extraction methods were mainly carried out using airborne laser scanning (ALS) data. Yang et al. implemented a marked point process method which extract building outlines from ALS point clouds [1]. Albers et al. [2] used an energy minimization approach for regularization on the initial results of building extraction to achieve a certain degree of optimization. Du et al. [3] combined point-based and grid-based features to obtain a promising result of building extraction on the entire ISPRS dataset. For the problem that buildings and vegetation are difficult to distinguish, Huang et al. [4] proposed a top-down strategy based on the object entity to achieve good performance. Compared with ALS data, more researchers have widely studied with mobile laser scanning (MLS)/terrestrial laser scanning (TLS) point clouds. For MLS point clouds, some people [5,6] tend to extract building based on the spatial distribution patterns, however, the performance of their results was restricted by the quality of data. Wang et al. [7] proposed an efficient method to highly extract building facade by combining the point clouds and optical images. Pu et al. [8] proposed a building facade recognition algorithm based on knowledge rules, but it has certain limitations and is difficult to apply to complex outdoor scenes. Attila et al. [9] used the high-difference feature to identify and extract building objects by dividing the grid. Xia et al. [10] proposed a “localization then segmentation” framework to solve several challenges and achieve instance-extraction of buildings from point clouds in residential areas. In summary, object-oriented methods are highly restricted by the performance of scene segmentation for identifying building components.

Thanks to the development of 3D semantic segmentation technology, the classification-based building extraction method was boomed in recent years. Specifically, discriminative features are extracted and then used to make inferences via an unsupervised strategy or supervised classifiers. Aijazi et al. [11] recognized building objects based on super-points with similar properties. Similarly, Wang et al. [12] proposed using voxel to replace point could and apply different rules to improve the identification of individual building objects. Yang et al. [13] suggested a method to generate multi-scale super-points from point clouds, and then merge meaningful targets according to specific rules. Niemeyer et al. [14] applied Conditional Random Field and features aggregation of different levels to recognize some objects including buildings. Zhu et al. [15] proposed a point cloud classification method with multi-level semantic relations, which uses multi-level context information to classify and extract building objects from LiDAR data. For the reason in which a large amount of prior information is used to obtain global consistency optimization results, so the applicability of the method is limited. Some deep learning methods [16,17,18,19,20] have also been applied, but these methods require a large number of training samples and are currently only implemented on the semantic 3D dataset [21].

At present, researches on extracting buildings based on point clouds are still insufficient. To address the issues raised from the state-of-the-art of the classification of laser scanning data, in this paper, instead of directly using low-level features, we carefully investigate the influence of several different feature sets on semantic labeling for building extraction. Super-points are treated as basic operational units in feature extraction considering its computational efficiency. These features are combined together to compose different feature sets which are further applied to the Random Forest classifier for classification. Besides, to consider more contextual information into semantic labeling, we optimize the super-point labels to generate locally continuous and globally optimal classification results by MRFs, which do not require fully supervised training scenes. This improves the labeling results by reducing unnecessary categories used in describing a region. Finally, based on label, the proposed hierarchical method extracts building from MLS/TLS data in urban and campus environments.

The remainder of this paper is organized as follows. Following the introduction, the key components of our proposed method are carefully illustrated in Section 2. In Section 3, the experimental studies and analysis are elaborated. Section 4 discussed the results of the experiments. The conclusion is given at the end of this paper.

## 2. Materials and Methods

**Figure 1:**
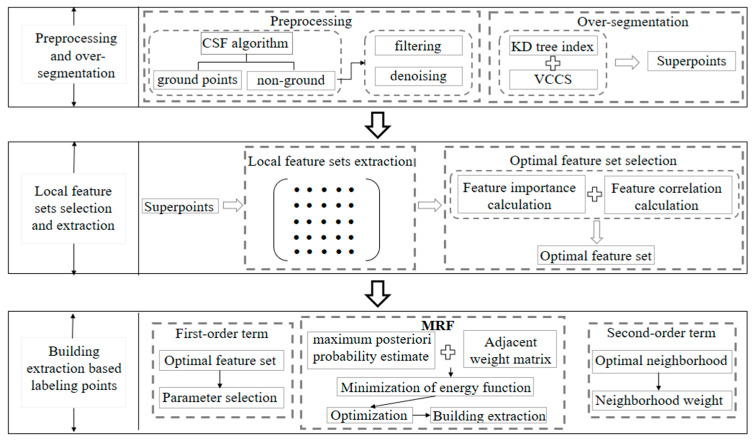
Overview of the proposed framework.

The proposed method is carried out according to a hierarchical process, the workflow is shown in Figure 1. The LiDAR point clouds were firstly identified as ground and off-ground points using an existing ground filtering algorithm [22] to eliminate the connectivity between different objects. And then, outlier and noise filtering are performed in off-ground points. The further process consists of three main steps:

1. Non-ground points are over-segmented to generate super-points;
2. Local feature sets selection and extraction;
3. Building extraction based on point cloud classification using context information.

Each step of our method detailed as follows.

### 2.1. Super-Points Generation of Non-Ground Points

First, the LiDAR raw point clouds are inverted, and the inverted surface then is cover by a rigid cloth. The locations of the cloth nodes are determined by analyzing the interactions between them and the corresponding points, which can generate an approximation of the ground surface. Finally, the ground points can be extracted from the LiDAR point cloud by comparing the original LiDAR points and the generated surface. After ground points are separated from the scene, spatially relatively independent non-ground points are obtained, but the amount of data is still huge. It is highly challenging to point-wise process, for example, a heavy computing cost. In order to improve the segmentation efficiency for large scale scenes and reduce the heavy burden of a large number of points, the proposed method divided raw scene space into super-points which was taken as basic units in further processing.

The super-points generation in the proposed method is different from other segmentation algorithms, in which points within each super-point have consistent geometric characteristics and appearance. Its purpose is to divide point cloud into smaller clusters, not to achieve a certain segment. The proposed method focuses on building extraction from LiDAR point clouds, and it is necessary to preserve object boundaries well. Several existing methods face a challenge due to the LiDAR point clouds with non-uniform density and is often overlap. VCCS (Voxel Cloud Connectivity Segmentation) algorithm [23] and its related methods [24,25,26,27] may not effectively preserve boundary information. In addition, some advanced algorithm [28], can preserve object boundaries and small structures more effectively, but it is likely to be sensitive to the data quality.

**Figure 2:**
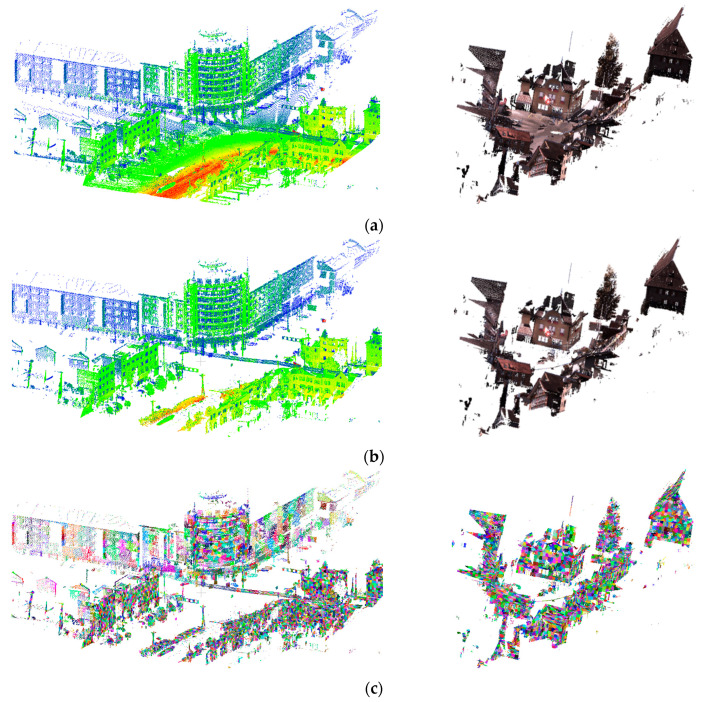
Super-points generated from own (**left**) and benchmark (**right**) datasets. (**a**) Raw data, (**b**) raw data without ground, (**c**) super-points results generated by our method.

To make super-points conform better to object boundaries and provide accurate geometric information for further processing, we replace the adjacency octree index in the VCCS algorithm with K-nearest neighbor to expand the super-points [29]. Unlike VCCS, which selects seeds with a unified resolution, the proposed method adopts the k-NN search to establish adjacencies between super-points by the neighboring relationships. Moreover, to benefit the preservation of more geometric features, the proposed method works directly on the original data instead of a voxelized point cloud. The super-points generated in the proposed method are adequately homogeneous and derive accurate local geometric information (as shown in Figure 2c). In this study, the features of a point in one super-point were calculated using all of these points in this super-point, meaning the features of all points within this super-point were the same, and all points were assigned the same class label within one super-point [30].

### 2.2. Local Feature Sets Selection and Extraction

All points within one super-point is assigned the same label, so these points are characterized by analogous properties. Super-point is treated as a basic operational unit, which means that different local features will be extracted based on the derived super-point neighborhood after over-segmentation. The biggest benefits are robustness to noise and outliers, and reduced computational cost. As an essential process in building extraction, point cloud classification needs to fully consider the local feature types of point clouds which can distinguish building objects. It also needs to ensure the consistency of buildings and extract building objects completely. Feature selection and extraction serve as the basis for 3D semantic segmentation. There is no doubt that their performance plays a decisive role in classification and subsequent processing.

As the most important man-made object in urban scenes, the building structure has obvious geometric features. After generating the super-points, we carefully selected some types of local features in this study: height, orientation, planar, covariance and projection features. These features described the differences between building and other objects in the scene in several ways. According to the geometric features of the clusters, we constructed a set of feature vectors for classification, as shown in Table 1.

**Figure 3:**
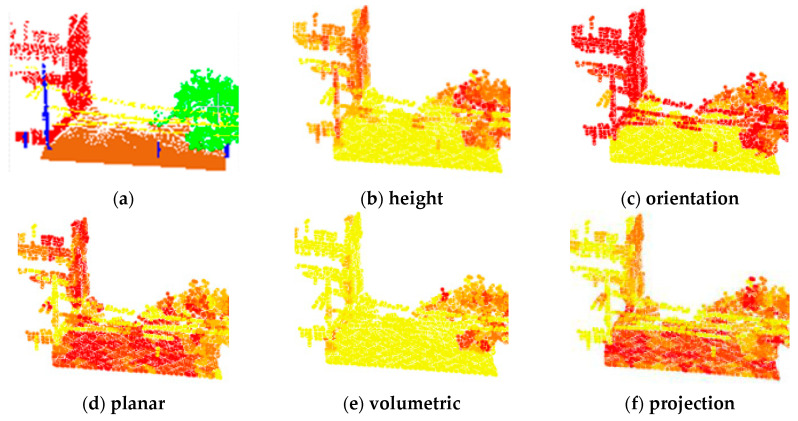
Different feature distribution. (**a**) Point cloud with label, (**b**–**f**) the value distribution of each feature in the area with color-coded feature values, where yellow represents a low and red a high value.

Local feature sets of point cloud can be written as $F=\left[D_{z},\sigma_{h},\lambda_{1},\lambda_{2},\lambda_{3},L_{\lambda },P_{\lambda },S_{\lambda },A_{\lambda },O_{\lambda },C_{\lambda },\theta ,D,PA_{h},PA_{v}\right]$, which consists of the normalized height $D_{z}$, elevation difference standard deviation $\sigma_{h}$ of the height feature, the covariance features (including eigenvalues $\lambda_{1},\lambda_{2},\lambda_{3}\left(\lambda_{1}\geq \lambda_{2}\geq \lambda_{3}>0\right)$; $L_{\lambda }=\lambda_{1}-\lambda_{2}/\lambda_{1}$, $P_{\lambda }=\lambda_{2}-\lambda_{3}/\lambda_{1}$, $S_{\lambda }=\lambda_{3}/\lambda_{1}$, which are the linear, planar, and volumetric geometric features; anisotropic feature $A_{\lambda }=\lambda_{1}-\lambda_{3}/\lambda_{1}$; curvature $C_{\lambda }=\lambda_{3}/\lambda_{1}+\lambda_{2}+\lambda_{3}$ and structural tensor change index $O_{\lambda }={\left(\prod_{i=1}^{3}\lambda_{i}\right)}^{1/3}$), local direction represented by the angle θ between normal vector of each superpoint and normal vector of the horizontal plane, planar geometric structures D and projection features $PA_{h}$ and $PA_{v}$. Different types of features have different saliences for different objects. A combination of features separates multiple objects in outdoor scene as much as possible. The heat map distribution of features in scene under different features is shown as Figure 3. It can be found that the height features are more prominent in buildings and trees, the orientation in buildings, roads and power lines, the planar features on buildings and the ground, and the volumetric features on trees. Significantly, the projection features are more prominent on the ground point cloud, which means that different features have a certain ability to distinguish special objects, so integrating multiple types of features will help distinguishing building from the scene.

Once a variety of local features of point clouds has been extracted, it has to be considered that there may be redundant or irrelevant information with respect to the semantic segmentation. Hence, it is often desirable to select a compact subset of relevant features that can achieve the best performance. The purpose of feature selection [31] is to remove features with weak classification ability, a significant increase of classification efficiency as well as accuracy can be expected due to much less involved information. The feature selection in proposed method mainly includes two steps: (1) Obtain and rank the importance index of each feature to the category by derived scores, and the lower ranking feature is considered to have a weak classification ability; (2) Calculate the correlation coefficient between the features. If the correlation coefficient between the two features is higher, the lower-ranked feature is considered to be a redundant feature and can be deleted. The feature combination obtained by setting the correlation coefficient threshold is the training feature vector set that the final point cloud classification depends on. To avoid a classifier-dependent solution for deriving feature subsets, we directly calculate relevance from training data by a multivariate filter-based feature selection [32] where evaluates intrinsic properties of the given data. The value of the feature can be regarded as continuous in a certain interval, we evaluate score function with respect to both feature-class and feature-feature relations. As relevant properties of the given data may be relevant for scene analysis, the correlation between the two continuous variables is calculated by several measures [33], such as, information gain (a measure revealing the dependence between a feature and a class label) [34] and Pearson correlation coefficient (a measure indicating the degree a feature is correlated with a class label) [35]. Following the provided implementation, a higher value indicates more relevance.

**Figure 4:**
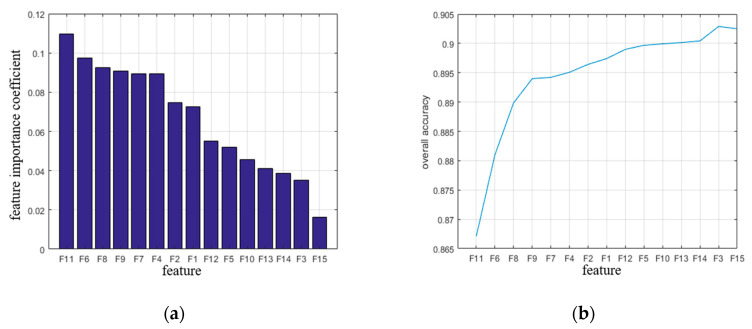
Relationship between feature correlation selection and classification accuracy (**a**) feature importance ranking; (**b**) relationship between feature selection and accuracy.

Figure 4a,b are the importance ranking of features and the effect of feature selection on classification accuracy, respectively. It can be found that F14 (horizontal projection feature $PA_{h}$), F3 (feature value $\lambda_{1}$) and F15 (minimum vertical projection feature $PA_{v}$) have the least importance, for this reason, we assume the three worst-ranked feature of importance metric to be pointless in the experiments. Following the principle of forward selection, we begin with only the most importance feature. Subsequently, the derived order of the features is used to successively train and test the classifiers with one additional feature per iteration. As shown in Figure 4b, the classification accuracy reaches the highest precision value of 0.903 after adding the $\lambda_{1}$ feature (F3) while the accuracy decreases after adding the $PA_{v}$ feature, indicating that the feature has an impact on the classification accuracy and can be deleted.

**Figure 5:**
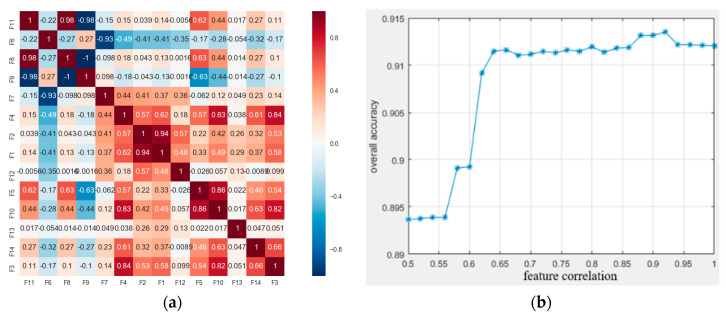
Relationship between feature correlation selection and classification accuracy. (**a**) Feature correlation heat map; (**b**) relationship between feature correlation and accuracy.

After deleting the minimum vertical projection feature by importance judgment, the relevance metric between features is calculated according to importance ranking, as shown in Figure 5a. Set the correlation threshold $c_{t}$ to 0.5~1, and judge relationship between correlation and classification accuracy under different thresholds, as shown in Figure 5b. In this paper, the feature where the pairwise correlation coefficient is greater than or equal to $c_{t}$ is considered as a candidate redundant feature and needs to be deleted. When classification accuracy reached the highest value of 0.913, its corresponding feature correlation is 0.92, so $c_{t}$ is set to 0.92. F11 (curvature feature $C_{\lambda }$) and F8 (spheroidal feature $S_{\lambda }$ based on eigenvalue), F1 (normalized height feature $D_{z}$) and F2 (height standard deviation feature $\sigma_{h}$) satisfy the condition. Furthermore, since the importance of $C_{\lambda }$ is greater than $S_{\lambda }$, the spherical feature $S_{\lambda }$ is deleted; similarly, the normalized height feature $D_{z}$ is discarded since the importance of $\sigma_{h}$ is greater than $D_{z}$.

Finally, after combining two constraints of feature importance and correlation, minimum vertical projection feature $PA_{v}$, eigenvalue-based divergence feature $S_{\lambda }$ and normalized height feature Δh are deleted, and the optimal feature set $\left[\sigma_{h},\lambda_{1},\lambda_{2},\lambda_{3},L_{\lambda },P_{\lambda },A_{\lambda },O_{\lambda },C_{\lambda },\theta ,D,PA_{h}\right]$ is obtained. Moreover, after removing redundant features, the classification accuracy improved from 0.903 to 0.913, which indicates that feature redundancy will affect the classification result.

### 2.3. Label Refinement by Higher Order MRF

The above features are scaled into a range [0, 1] before being applied to the classifier. To recognize candidate objects from complex environment, a Random Forest (RF) classifier was used for point cloud classification. Specifically, the classifier was trained on manually labeled data; the proposed method classifies the entire scene through a trained RF classifier. Unfortunately, using only local features in prone to label noise, which means that the classification results lack consistency. We consider more context information to optimize the results. The MRFs can describe the relationship and interactions among adjacent data and are used to perform spatial context construction.

We formalize the solution of point cloud optimal classification label configuration as the maximum posteriori probability estimation problem of MRFs. Inspired by the work of computer vision, this problem can be naturally formulated in terms of energy function minimization which is designed as follows:

(1) $$L^{*}=argmin\left(E_{data}\left(L\right)+\lambda *E_{smooth}\left(L\right)\right)$$

where, $E_{data}\left(L\right)$ is first-order data term which measures the disagreement between label and raw data, while second-order smooth term $E_{smooth}\left(L\right)$ mainly describe the inconsistency of labels in local neighborhoods based on local context information; λ is the weight coefficient between the first-order potential and the second-order potential. In this paper, the point cloud classification results are obtained by solving the minimized energy function.

Local neighborhood construction is the most important part in point cloud classification optimization based on MRF model, which is beneficial to create context relationships among local point clusters. In the existing MRF model, the local neighbor system is created by using the K-nearest neighbor, and the K-clusters with the closest spatial distance are clustered into a neighboring system. However, since only the spatial distance is considered, this method tends to propagate optimization errors in overlapping occlusion regions (for example, at the intersection of buildings and trees, it is easy for partially overlapping buildings to be optimized into tree types).

**Figure 6:**
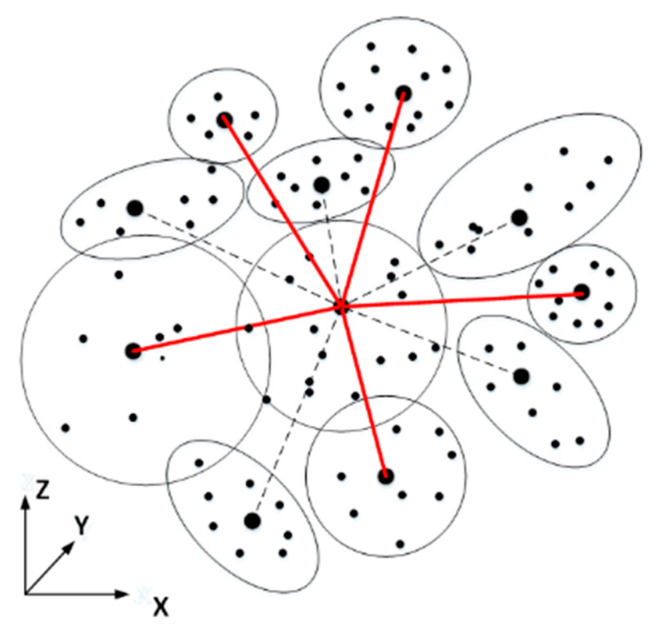
Schematic diagram of optimal neighborhood system.

In order to solve the problem of classification optimization error propagation, the similarity relationship among clusters is calculated and clusters with high similarity are selected to construct a local optimal neighborhood system, as shown in Figure 6 (The red line connection constitutes the optimal neighborhood system, indicating the point cluster with higher similarity; dotted line connects the dissimilar point clusters need to be deleted from the neighborhood during the construction process). The proposed method is based on the obtained optimal local feature set, and then selects from K-nearest neighbors the clusters whose correlation satisfies the threshold *p* < 0.70 to construct an optimal neighborhood system.

The probability distribution problem is transformed into the energy function problem, and then the optimal solution of the point cloud classification is obtained by minimizing the energy function. Minimizing the energy function is an NP-hard problem, and most state-of-the-art methods (e.g., Iterated Conditional Model and Simulated Annealing) achieve quite good results in terms of solution quality. However, for large-scale point clouds, using larger values of K will still bring a huge computational burden. For the classical algorithm, it needs to go through multiple iterations of small changes each time and the calculation efficiency is low. In this paper, the graph cut algorithm [36] is used to minimize the energy function. This method can make more changes to the label each iteration and reduce the number of iterations to achieve efficient energy optimization calculation.

The calculation of the energy function mainly includes first-order and second-order term. The first-order energy function mainly measures the inconsistency between the prediction and ground truth under a given feature set *F*. In this paper, the Random Forest algorithm (RF) is used to represent the energy function according to the posterior probability estimation of the local optimal feature, i.e.,

(2) $$E_{data}\left(L\right)=\sum_{i\epsilon P}D\left(l_{i}\right)$$

(3) $$D\left(l_{i}\right)=p\left(l_{i}=c_{i}|f\left(p_{i}\right)\right)=\frac{N_{c_{i}}}{N_{T}},\;\left(c_{i}\epsilon C=\left\{c_{1},c_{2},\cdots c_{m}\right\}\right)$$

where, $N_{c_{i}}$ is the number of votes for each class $c_{i}$, and $N_{T}$ is the number of weak classifiers of RF. In this paper, 200 is selected through cross-validation.

The weight of the adjacent edge is calculated according to the adjacency relationship, and then the second-order energy function is calculated. The calculation formula is as follows:

(4) $$E_{smooth}\left(L\right)=\sum_{i,j\in N}V_{i,j}\left(l_{i},l_{j}\right)$$

(5) $$V_{i,j}\left(l_{i},l_{j}\right)=w_{ij}*\delta \left(l_{i},l_{j}\right)$$

(6) $$w_{ij}=e^{-{\left(\frac{d\left(i,j\right)}{\sigma }\right)}^{2}}$$

(7) $$\delta \left(l_{i},l_{j}\right)=\{\begin{array}{c} 1\;if\;l_{i}\neq l_{j}\\ 0\;if\;l_{i}=l_{j} \end{array}$$

where, d(i,j) is the Euclidean distance of the cluster centroid, and σ represents the average of the spatial distance.

**Figure 7:**
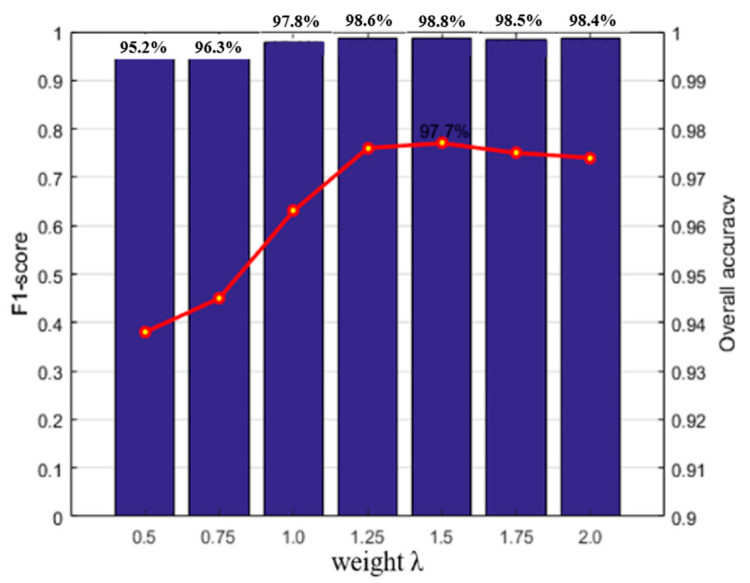
Impact of the weight λ on semantic labeling results.

In order to choose the optimal weight *λ* which is the coefficient of equilibrium data term and smooth data, we analyzed the impact of *λ* on the performance of labeling on Dataset A. The weights *λ* are set to 0.5, 0.75, 1.0, 1.25, 1.5, 1.75 and 2, respectively. As shown in Figure 7, the initial labeling results of buildings improves with the changes of the parameter *λ*. When the smoothing factor reaches 1.25, the F1-measure of the building tends to be stable, and the classification accuracy peak value reaches at *λ* = 1.5. Larger weight value means more costs imposed on the number of used categories, however, may lead to over-smooth results for labeling point clouds. Whereas a smaller *λ* means less penalty for the number of categories used in the region, which will result in a relatively large quantity of incorrect labels that cannot be effectively corrected. Properly setting the smoothing term coefficient to 1.5 can achieve balance and get the highest building classification accuracy, thereby obtaining promising fine labeling results.

**Figure 8:**
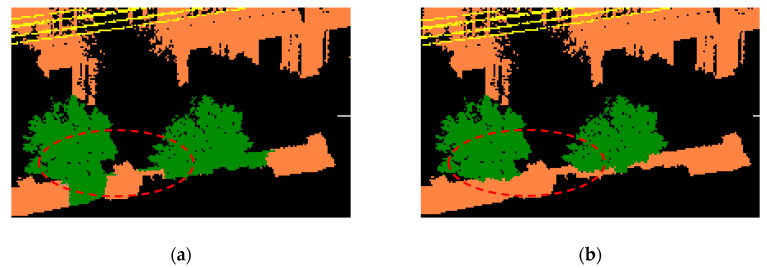
Classification results after optimization. (**a**) results after ordinary K-nearest neighbor optimization; (**b**) results after optimized K-nearest neighborhood optimization.

The initial label is adjusted by the *α*-expansion algorithm [37], which mainly merges the wrong categories into the majority of the surrounding classes, thereby reducing the inconsistency of the local classification. The minimum energy function was solved by a graph cut algorithm to obtain optimized classification result. Then in order to compare the effects of the optimized neighborhood system, this paper also works under the ordinary neighborhood system and compares the two classification results, as shown in Figure 8. It is easy to find that the result based on K-nearest neighbors (as shown in Figure 8a) show that neighboring objects can easily cause error propagation in the occlusion area due to the method only considers the spatial distance and ignores the similarity between different types of objects. Because the optimized neighborhood system considers the similarity of local clusters, the classification results, can effectively avoid the propagation of the optimization errors at intersections.

### 2.4. Building Extraction Based on Semantic Labels

The point clouds labeled as a building are extracted from the classification result of the scene. In order to obtain complete and independent building objects, clusters are merged into a single object according to connectivity relationship. Small clusters with less than 20 points are deleted to filter out then the points that are misclassified as buildings. The specific process is as follows: (1) extract a point set *C* marked as a building from the scene classification results; (2) select cluster $C_{i}$ are selected from *C*, and obtain $C_{j}$ based on the 4-NN search; then iterate all candidate clusters and determine whether the distance between $C_{i}$ and neighbor cluster $C_{j}$ is the Equation (8); if it does, two clusters can be merged, and the cluster $C_{j}$ is set to be clustered, otherwise $C_{j}$ was deleted; (3) If no new clusters were added, then a building object was clustered; (4) this process is repeated until all cluster are labeled to be completed. The building is eventually extracted.

(8) $$\left|C_{i}-C_{j}\right|\leq D_{threshold}$$

where, $D_{threshold}=\left|C_{i}+C_{j}\right|$ indicates the size of the combination of two clusters.

## 3. Results

LiDAR data from three different complex and challenging scenes are used for qualitative and quantitative evaluation and analysis to verify the performance of the proposed framework optimization method. The experimental datasets are firstly introduced, in this section, then the proposed method is validated in experimental studies to present and analyze results with just mentioned datasets.

### 3.1. Experimental Data Description

To check the performance of the presented framework on LiDAR point clouds, we performed both qualitative and quantitative evaluations on three different data sets. The point clouds in dataset A are part of the urban scene in Hengdian, Zhejiang, China, collected through the SSW-MMTS mobile mapping system. As described in [13,38], the SSW-MMTS mobile mapping system integrates a laser scanner with a maximum range of 300 m, a navigation and positioning system, and six high-resolution digital cameras (22 million pixels each), installed on the roof of a minivan. The point density of the points in this area is about 77 points/m^2^. Dataset B was captured around urban and rural outdoor scenes in Zurich, Switzerland with 30 static terrestrial laser scanners, [21,39] explains the large-scale 3D outdoor benchmark datasets, having point number about 600 million 3D points with different densities and colorizing from camera image. Dataset C [40] is a total of about 8 million points acquired around Wuhan University campus in Wuhan, Hubei, China using SICK LMS291 Laser Scanner; the dataset has a low point density and belongs to a low-resolution laser scanning data. The collected point clouds of dataset C without color information due to lack of digital cameras and the amount of points is quite smaller than those in dataset A and B. In the above three different datasets, many objects are often incomplete due to mutual occlusion, which is extremely challenging. Our team and other collaborators carefully classified all points with the CloudCompare (http://www.cloudcompare.org/) tool to evaluate the performance of the proposed framework. All datasets are divided into training samples for learning procedure and testing samples for the evaluating the performance of the proposed methods.

### 3.2. Experimental Results

#### 3.2.1. Preliminary Results of Semantic Labeling Using Contexts and MRF-Based Optimization

**Figure 9:**
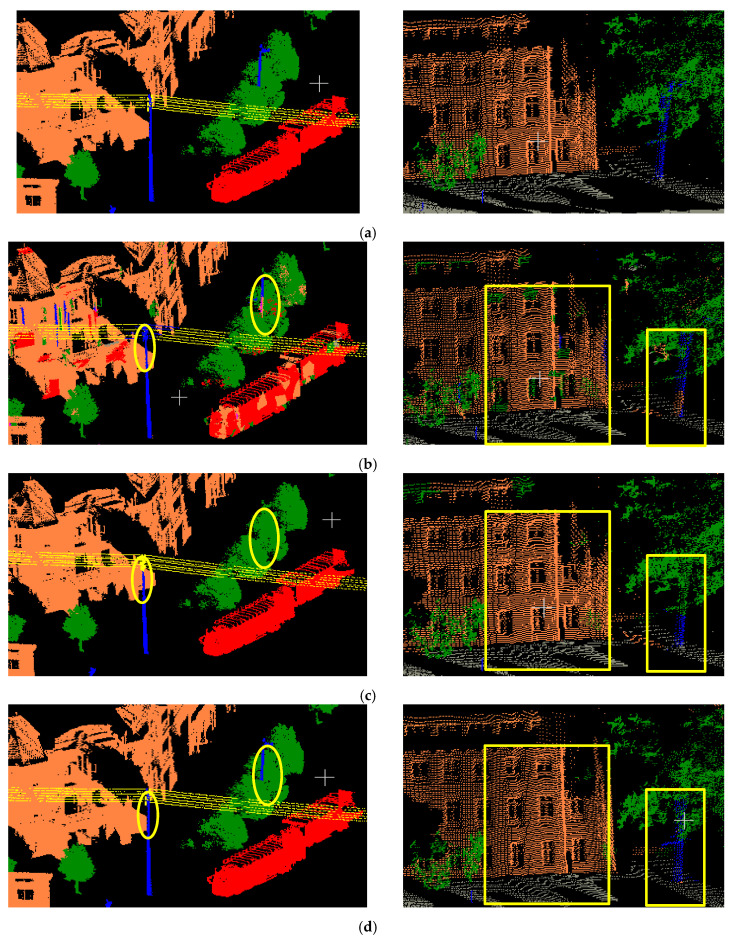
The initial scene semantic labeling and the comparison with the spatially smoothed results for selected point clouds.

During the learning phase, manually labeled points are used as input to train the RF classifier at each iteration. The number of decision trees and the depth of each tree in the RF are set at 100 and 15, respectively. The initial scene semantic labeling and the comparison with the spatially smoothed results of buildings (rendered with yellow color) for selected point clouds test data are shown in Figure 9. Figure 9a show the ground truths and are colored according to the labels of each point. Illustration of the initial semantic segmentation results of candidate objects are provided in Figure 9b, a small part of which are mislabeled points caused by local feature similarities, e.g., generally incomplete building façade incorrectly identified as trees. Figure 9 show the results of MRF classification optimization based on ordinary K-nearest neighbors. In Figure 9d, the spatially smoothed results of MRF classification based on optimized neighborhood are given.

#### 3.2.2. Classification-Based Extraction of Buildings

**Figure 10:**
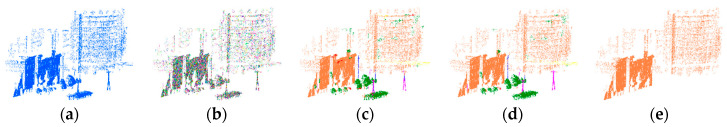
The illustration of building extraction of the purposed method. (**a**) Raw data; (**b**) super-points of non-ground points; (**c**) the initial results of semantic segmentation; (**d**) the final results of semantic segmentation; (**e**) the results of building extraction.

**Figure 11:**
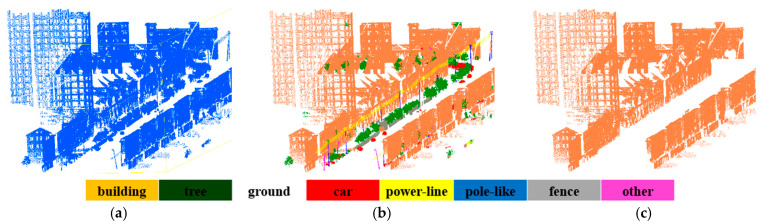
The results of building extraction on dataset A. (**a**) Raw data; (**b**) the final results of semantic segmentation; (**c**) the results of building extraction.

**Figure 12:**
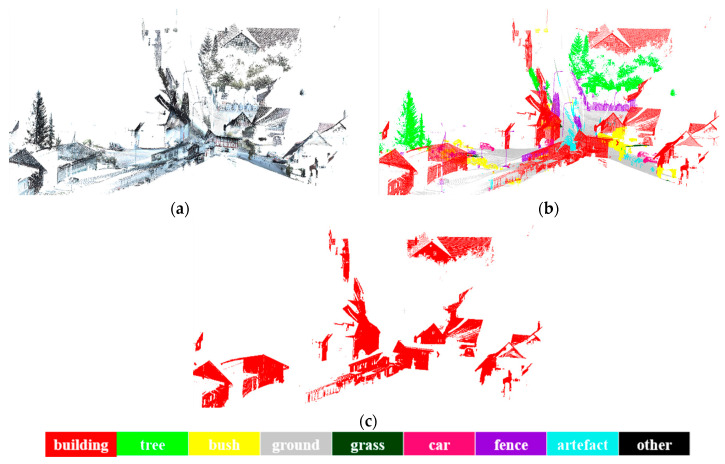
The results of building extraction on dataset B. (**a**) Raw data; (**b**) the final results of semantic segmentation; (**c**) the results of building extraction.

**Figure 13:**
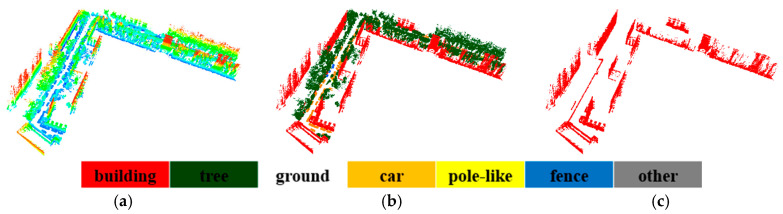
The results of building extraction on dataset C. (**a**) Raw data; (**b**) the final results of semantic segmentation; (**c**) the results of building extraction.

After labeled the points of buildings, a classification-based segmentation is performed to extract all the buildings. The illustration of segmentation of buildings is given in Figure 10, urban environment is taken as an example, and the proposed method can effectively work for building objects extraction. However, due to the complexity of the datasets, some points are difficult to distinguish and mistakenly segmented. Figure 11, Figure 12 and Figure 13 is the results of building extraction on dataset A, B and C.

### 3.3. Experimental Analysis

To quantitatively evaluate the performance of the proposed method for semantic labeling and recognizing buildings on these three data sets, four evaluation indexes were adopted in this study. The recall represents the percentage of completeness, while precision means the percentage of exactness. The overall accuracy (OA) reflects the overall performance on the test set, and the $F_{1}$ score were specifically used to evaluate the classification performance on each single class. They were defined as follows:

(9) $$\mathrm{Precision}=\frac{TP}{TP+FP}$$

(10) $$\mathrm{Recall}=\frac{TP}{TP+FN}$$

(11) $$F_{1}=\frac{2\times \mathrm{Precision}\times \mathrm{Recall}}{\mathrm{Precision}+\mathrm{Recall}}$$

(12) $$\mathrm{OA}=\frac{TP+TN}{TP+FP+FN+TN}$$

where *TP* (true positive) denotes the number of objects labeled with correct classes; *FP* (false positive) represents the number of objects which are recognized, but not in the corresponding reference set; and *FN* (false negative) is the number of incorrectly classified objects; *TN* (true negative) is the number of negative samples that are correctly classified as negative [41].

Table 2 and Table 3 show the results of quantitative analysis of K-nearest neighbors and optimal neighborhoods of these three data sets. On these three datasets, the classification accuracy of building objects in optimal neighborhood-based optimization is approximately 0.8%, 1.1% and 1.4% higher than that based on K-nearest neighbors, respectively. This further illustrated that the optimal neighborhood system has capability to handle the incompleteness and occlusion by considering the long-range contexts. In the light of buildings have a large proportion in the entire scene, the improvement of building classification accuracy has a significant impact on the classification accuracy of the scene. The proposed method (optimization based on optimal neighborhood) achieves good performance with overall accuracy of 95.9%, 94.3% and 84.7% for the three data sets, respectively. In particular, the results of classified buildings are well-pleasing.

Major parts of our method were implemented in C++ except that the semantic label and building extraction stage were finished using Python. Point Cloud Library [42], OpenCV [43] and Scikit-learn [44] are used in our program. Table 4 lists the processing time costs for each stage of our method. These results show that most of the total time was costed on generation of super-points on each dataset, because this step is a point-based process. Positively, the efficiency of subsequent processing is greatly improved thanks to super-points is used as the basic units. 

### 3.4. Comparative Studies

To further demonstrate superiority of the proposed method, it is compared with the previous studies of [13,40,45] in terms of overall accuracy of semantic labeling for entire scene. In particular, the building extraction is highlighted as listed in Table 5. We compared the results of semantic labeling and construction extraction on dataset A with other recent methods (Yang et al. [13] and Zhang et al. [45]). Overall, the accuracy of our method reaches 95.9%, which is much higher than the latter. On dataset B, we compared the proposed method with two following works: Yang et al. [13] and Zhang et al. [45], and the proposed method also achieved the highest accuracy for semantic segmentation and classification accuracy reached 95.4%, which was slightly higher than the results achieved by other two methods. For sure, for all of the comparison methods, building extraction has obtained satisfactory results. For point cloud classification on dataset C, some other methods, including Yang et al. [13] and Wang et al. [40] are compared with the proposed method. It is noted that our proposed method achieves the best results of objects recognition and building extraction.

## 4. Conclusions

This paper has presented a method for effectively conducting semantic labeling and building extraction from LiDAR point cloud, including (1) separating the ground and non-ground points using an advanced existing filtering approach; (2) generating spatially consistent super-points, rather than individual points, are generated from non-ground points; (3) extracting different features based on the super-points neighborhood and selecting some optimal features then using them for point classification; (4) obtaining the initial semantic labeling results using the random forest classifier and refining the initial results based on optimized neighborhood by considering more contexts; (5) building extracting according to the semantic labeling results. The main contributions of proposed approach are as follows: non-ground points are over-segmented to generate super-points to improve the estimation of local geometric features of neighboring points and the segmentation efficiency; select local feature sets for semantic segmentation to remove features with weak classification ability and achieve the best performance of building extraction; design a MRF model introduced high-order contextual information for the refinement of the classification; a hierarchical segmentation strategy is robust to noise and situation of occlusion and overlapping. Experiments on three different datasets prove that this method had good applicability for building extraction from point cloud in complex environments.

Future work will be refined in the following aspects: reducing the number of manual parameters in our proposed model effectively to further strengthen the generalization ability; generating multiscale super-points toward better boundary and preserving small structures in an effective way to cut down time cost; considering more multiple levels features and contextual features to enhance descriptiveness; using a higher MRF model to optimize results of semantic segmentation by taking into account the long-range contexts among local variables; extracting buildings directly from the scene and performing instance segmentation.

## Figures and Tables

**Table 1:** Feature vectors set for classification.

	Local Features	Descriptors	Dimension	Identifiable Objects
1	Height feature	$D_{z}$	2	power line
2		$\sigma_{h}$		
3	Covariance matrix feature	$\lambda_{1}$	9	building, power line
4		$\lambda_{2}$		building
5		$\lambda_{3}$		tree
6		$L_{\lambda }$		building, power line
7		$P_{\lambda }$		building
8		$S_{\lambda }$		tree
9		$A_{\lambda }$		tree
10		$O_{\lambda }$		tree
11		$C_{\lambda }$		building
12	Angle feature	θ	1	building façade, tree
13	Planarity feature	*D*	1	building façade, tree
14	Projection feature	$PA_{h}$	2	building façade, pole
15		$PA_{v}$		pole-like

**Table 2:** Quantitative spatially smoothed results based on K-nearest neighbor. (Precision (P), Recall (R), $F_{1}$ score (F1)).

	Dataset A			Dataset B			Dataset C		
	P	R	F1	P	R	F1	P	R	F1
Buildings	96.9%	97.6%	97.2%	94.3%	98.6%	96.4%	93.2%	92.1%	92.5%
Trees	88.1%	94.1%	91.0%	84.7%	98.6%	91.1%	85.8%	94.2%	89.1%
Bush	/	/	/	94.1%	46.0%	61.8%	/	/	/
Pole-like	84.4%	79.4%	81.8%	/	/	/	27.9%	32.4%	30.1%
Ground	98.9%	99.1%	99.0%	98.8%	98.5%	98.6%	99.1%	97.2%	98.1%
Grass	/	/	/	94.1%	96.5%	95.3%	/	/	/
Powerline	84.6%	85.4%	85.0%	/	/	/	/	/	/
Cars	81.5%	86.8%	84.1%	83.4%	83.4%	83.4%	57.6%	95.2%	60.9%
Fence	93.2%	96.5%	95.4%	10.4%	4.4%%	6.2%	99.8%	60.9%	87.1%
Artefacts	/	/	/	52.9%	74.7%	61.9%	/	/	/
Others	90.9%	91.5%	91.2%	88.6%	89.3%	88.9%	15.9%	1.9%	2.8%
OA	93.2%			93.2%			83.3%		

**Table 3:** Quantitative spatially smoothed results based on optimal neighborhood. (Precision (P), Recall (R), $F_{1}$ score (F1)).

	Dataset A			Dataset B			Dataset C		
	P	R	F1	P	R	F1	P	R	F1
Buildings	97.7%	97.5%	97.5%	95.4%	98.5%	96.9%	94.6%	93.5%	92.9%
Trees	94.8%	93.7%	93.8%	84.9%	98.5%	91.2%	89.1%	95.0%	93.4%
Bush	/	/	/	94.2%	50.3%	65.6%	/	/	/
Pole-like	84.9%	88.1%	86.5%	/	/	/	85.6%	40.6%	79.0%
Ground	99.5%	99.1%	99.3%	98.9%	98.6%	98.7%	98.4%	98.8%	98.6%
Grass	/	/	/	94.4%	96.7%	95.5%	/	/	/
Powerline	91.2%	92.2%	93.3%	/	/	/	/	/	/
Cars	94.5%	92.2%	93.3%	85.6%	86.2%	85.9%	56.7%	90.3%	70.1%
Fence	93.5%	95.6%	94.7%	13.8%	7.9%%	10.0%	94.1%	61.8%	88.2%
Artefacts	/	/	/	62.8%	73.5%	67.7%	/	/	/
Others	89.5%	90.8%	90.5%	87.9%	89.8%	88.8%	8.9%	0.5%	1.4%
OA	95.9%			94.1%			84.7%		

**Table 4:** Time performance by each stage of the proposed method (s).

Dataset	Super-Points Generation	Features Computation	Initial Classification	Optimized Results	Buildings Extraction	Total Time Cost
A	859.543	106.425	2.343	3.194	20.161	1188.909
B	Total: 513.35 min					
C	305.532	46.317	0.520	1.672	8.413	375.145

**Table 5:** Performance comparison between the proposed method and others.

Dataset	Yang et al. [13]		Zhang et al. [45]		Wang et al. [40]		Proposed Method	
	Overall	Building	Overall	Building	Overall	Building	Overall	Building
A	92.3%	97.5%	90.6%	91.4%	/	/	95.9%	97.7%
B	85.3%	94.5%	93.2%	94.2%	/	/	94.1%	95.4%
C	82.9%	86.4%	/	/	83.2%	93.1%	84.7%	94.6%
